# Legal, professional, and practical aspects of psychotherapy education of psychiatrists in Germany

**DOI:** 10.1192/j.eurpsy.2025.168

**Published:** 2025-08-26

**Authors:** M. Linden

**Affiliations:** Department of Psychosomatic Medicine, Charité University Medicine Berlin, Berlin, Germany

## Abstract

**Abstract:**

In Germany there are about 40.000 licensed psychological psychotherapists, 4.000 physicians who work as psychotherapists only, 8.000 psychiatrists who also are specialised in psychotherapy, 4.000 physicians specialised in psychosomatic medicine and psychotherapy, 1.000 physicians of child psychiatry, 35.000 somatic physicians with a training in psychosomatic basic care. This accounts for about 1 therapist for 1.000 inhabitants or 1 per 200 persons with mental problems in ambulatory care. All these therapists are fully reimbursed by health insurance. About 60% of all persons with mental problems have been treated in specialised outpatient psychotherapy.

There are furthermore 900.000 patients per year who are treated for 30 days on average in inpatient departments of psychiatry, psychosomatic medicine and psychotherapy, and another 30.000 patients who are treated in about 300 psychosomatic rehabilitation hospitals for about four weeks.

Given these numbers and costs for psychotherapy, there are multiple regulations for the education and professional practice of therapists. Therapists must undergo three years of training either in cognitive behavior therapy or psychodynamic psychotherapy or systemic psychotherapy, while other forms of psychotherapy, such as Gestalt or Logotherapy are not allowed in training nor patient care. There are detailed requirements in regard to the number of theory lessons, therapeutic self-experience, and treatment with qualified supervision after four sessions. Psychotherapy practice is also restricted to these **“**scientifically accepted psychotherapies**”**, which is overseen by two state committees. Depending on the psychotherapy school, health insurance reimburses up to 36 sessions for systemic psychotherapy, 80 for cognitive behavior therapy, 100 for psychodynamic psychotherapy and 300 for analytical psychotherapy.

In summary, psychotherapy in Germany is part of regular patient care and therefore submitted to all respective regulations.

**Disclosure of Interest:**

None Declared

